# The process of incorporating insulin pumps into the everyday lives of people with Type 1 diabetes: A critical interpretive synthesis

**DOI:** 10.1111/hex.12666

**Published:** 2018-02-08

**Authors:** Claire Reidy, Mike Bracher, Claire Foster, Ivaylo Vassilev, Anne Rogers

**Affiliations:** ^1^ Faculty of Health Sciences NIHR Collaboration for Leadership in Applied Health Research (CLAHRC) Wessex University of Southampton Southampton Hampshire UK; ^2^ School of Health and Social Care Health Sciences Bournemouth University Bournemouth Dorset UK; ^3^ Faculty of Health Sciences University of Southampton Southampton Hampshire UK; ^4^ Faculty of Health Sciences Macmillan Survivorship Research Group University of Southampton Southampton Hampshire UK

**Keywords:** critical interpretive synthesis, insulin pumps, new technology, psychosocial, social support networks, Type 1 diabetes

## Abstract

**Background:**

Insulin pump therapy (IPT) is a technological advancement that has been developed to help people manage Type 1 diabetes (T1D). However, ways of managing diabetes requiring the implementation of health technologies bring new complexities and a need to understand the factors which enable people with T1D to incorporate a novel device. This new comprehension could provide an exemplar for people with long‐term conditions to incorporate new technologies more generally.

**Objective:**

To determine what influences the incorporation, adaptation and use of IPT into the everyday lives of people living with diabetes.

**Design:**

Critical interpretive synthesis (CIS) using systematic searches undertaken in 7 electronic databases of literature, published 2008 onwards.

**Results:**

A total of 4998 titles were identified, 274 abstracts reviewed, 39 full articles retrieved and 22 papers selected for analysis. Three themes emerged which were of relevance to the introduction and use of IPT; Tensions between expectations and experiences in adoption and early adaptation; Negotiation of responsibility and accessing support; Reflexivity, active experimentation and feedback.

**Conclusions:**

This CIS builds on earlier reviews on lived experiences of IPT. Novel insights are offered through examination of the experiences of pump users from children through to adults, their families and health‐care professionals. Expectations of what the device can do to improve self‐management impacts on the early stages of adoption as the reality of the technology requires substantial thought and action. Areas for intervention to improve IPT incorporation include establishing who is responsible for management tasks of the device and enabling navigation to further means of support and resources.

## 1 INTRODUCTION

Over 4 million people live with diabetes in the UK, and Type 1 diabetes (T1D) accounts for about 10% of that population.^1^ Continuous subcutaneous insulin infusion (CSII), insulin pump therapy, is a technological advancement used to support people with T1D manage their diabetes optimally. It is associated with psychosocial benefits (quality of life—QoL)^2^, ^3^, ^4^, ^5^, ^6^, ^7^, ^8^, ^9^, ^10^, ^11^, ^12^, ^13^, ^14^, ^15^, ^16^ and improved biomedical outcomes.^2^, ^4^, ^8^, ^10^, ^12^, ^14^, ^17^, ^18^, ^19^, ^20^, ^21^, ^22^, ^23^, ^24^, ^25^, ^26^, ^27^, ^28^, ^29^, ^30^, ^31^, ^32^, ^33^, ^34^, ^35^, ^36^, ^37^, ^38^, ^39^, ^40^, ^41^ Historically, new ways of managing diabetes through implementing new health innovations have brought new complexities and are of particular relevance to CSII, which is more technologically advanced than previous modes of insulin delivery. Understanding the impact of these advancements is an important avenue for exploration in providing a model of how people incorporate new and complex health tools which ostensibly provide much needed flexibility and choice in how people living with a long‐term condition(s) can self‐manage. The purpose of this review is to analyse existing literature about the processes of adoption, adaptation and embedding of a new physical health innovation (CSII) in the lives of people with T1D and the resources and support that enable this (Box 1).

Box 1 1 Insulin pumps are electronic devices, about the size of a pager, which drip feed rapid‐acting insulin via a fine cannula implanted into subcutaneous tissue continually throughout the day (called a basal dose).^47^ This device must therefore be worn constantly. The user then self‐administers, as required, extra shots of insulin (called bolus doses) to match their intake of glucose (carbohydrates) throughout the day. These extra doses of insulin can be much more specific (and minute) at delivering insulin than traditional insulin injections. This apparatus also integrates what is called a “bolus calculator/advisor/wizard,” which recommend an appropriate (and usually personalised) insulin dose to the user.

Optimal self‐care practices of people living with T1D constitutes a demanding and multifaceted regimen^42^ including monitoring and controlling blood glucose levels, which are subject to extreme fluctuations, and risk of complications (Box 2).^43^, ^44^, ^45^ While multiple daily insulin injections (MDI) remain the main delivery method of insulin therapy globally,^46^ both MDI and CSII are recommended.^38^, ^47^, ^48^ However, the focus of insulin delivery is shifting towards the latter as a method considered more physiologically representative of a fully functioning pancreas.^49^, ^50^ CSII has been shown to yield particular benefits over MDI,^16^, ^29^, ^38^, ^51^, ^52^ for example, lower cardiovascular mortality,^53^ higher treatment satisfaction^54^ and improved glucose control.^47^ In 2008, the National Institute for Health and Clinical Excellence (NICE) recommended CSII for people with T1D whose glucose levels were not well controlled by MDI.^38^ This has been estimated to apply to 15%‐20% of adults living with T1D in the UK,^55^ compared to 6% currently utilizing CSII.^56^

Box 2 1 “The [NICE] guidance states that continuous subcutaneous insulin infusion (CSII) or ‘insulin pump’ therapy is recommended as a treatment option for adults and children 12 years and over with Type 1 diabetes mellitus if:

- attempts to reach target haemoglobin A1c (HbA1c) levels with multiple daily injections result in the person having ‘disabling hypoglycaemia’, or
- HbA1c levels have remained high (69 mmol (8.5%) or above) with multiple daily injections (including using long‐acting insulin analogues if appropriate) despite the person and/or their carer carefully trying to manage their diabetes

CSII therapy is not recommended as treatment for people with Type 2 diabetes mellitus.”^102^

Two reviews of CSII in 2003 and 2009 found that while CSII improves glycaemic control, few studies have robustly assessed psychosocial aspects of using insulin pumps.^22^, ^27^ The latter is likely to be pertinent to assess given that CSII requirements are likely to constitute an added burden for people, particularly in the initial stages of adoption.^22^ A review by Barnard et al in 57 established that studies, which do measure psychosocial aspects of CSII, were characterized by; variable methodology and psychosocial constructs, small sample sizes, a focus on one particular patient group, or were dated (the devices have since become smaller, more accurate and more widespread). While understanding psychosocial outcomes is relevant to assessing the impact of CSII on well‐being,^22^, ^27^ it is important to explore the factors that may promote or inhibit its adoption and embedding as a self‐management strategy for T1D. Thus, exploration of existing evidence is needed to illuminate the processes and outcomes by which CSII becomes part of the management of diabetes.

This review is designed to build on earlier reviews by providing a current and in‐depth exploration of user experience, and those integrally involved in or impacting on this experience (i.e parents/caregivers/health‐care professionals [HCPs]). The aim is to offer enhanced understanding of mechanisms that shape the incorporation, adaptation and use of CSII into the everyday lives of people living with diabetes, and establish what support and resources are needed to enable this.

## 2 METHODS

### 2.1 Study design

Critical interpretative synthesis (CIS) was used to identify domains from the literature that are key to successfully incorporating CSII. CIS is an exploratory method of reviewing literature, focused on prioritizing generation of theory in synthesizing findings. CIS allowed us to pragmatically explore the range of data and understand factors which may enable someone to incorporate an insulin pump into their everyday lives. The review had 3 stages: (i) Systematic search, (ii) Critical appraisal and (iii) Synthesis.

### 2.2 Identifying relevant studies

A search strategy was developed incorporating the 3 main research aims: T1D (population); CSII (intervention); and terms relating to the psychosocial outcomes of the studies searched, using the PICOS model (Table 1). Different combinations of terms for each component were searched for (including relevant acronyms and truncations), to maximize capture of relevant literature.^58^ A systematic search of studies reporting users of pumps/HCP or significant other experiences of living with CSII was conducted using a range of databases: AMED; CINAHL; EMBASE; MEDLINE; PsycINFO; Cochrane database; Web of Science. An academic librarian and 3 other researchers (AR, MB and MCP) provided feedback on development of the search strategy and its results.

**Table 1:** Search strategy key terms

Number	Term	OR/AND
S1	“insulin pump”	OR
	“continuous subcutaneous insulin infusion”	
	“CSII”	
	“closed‐loop glucose control”	
	“Open‐loop glucose control”	
S2	Habituation*	OR
	Psychophysiologic*	
	Adaptation*	
	“Quality of Life”	
	“Normalisation”	
	“Normalization”	
	Incorporat*	
	Integrat*	
	Impact*	
	Perception*	
	Experience*	
	Opinion*	
	Attitude*	
	“Social‐support”	
	Cope*	
	Coping*	
	Burden*	
	“living with”	
	“psychosocial”	
	Psychol*	
	“Social‐functioning”	
S3	S1, S2	AND
S4	S3	Limited to English

### 2.3 Study selection and appraisal

Inclusion/exclusion (Table 2) and eligibility criteria (Table 3) were established using the PICOS approach. Initially, search criteria did not exclude studies based on publication date; however, early searches indicated that the (most recent) changes to NICE guidelines (2008)^38^ considerably widened pump uptake, and consequently technological advancement and research of this device. We therefore restricted our inclusion criteria to studies published 2008 onwards. However, some of the included papers were retrospective, and involved interviewing people who had been using CSII for 5+ years. These papers were included on the basis that they provided useful background and contextual information, and some of the barriers and facilitators to adoption and embedding of CSII remain relevant. Although quantitative evidence was also reviewed, these papers were not included in the final analysis because they did not sufficiently explore lived experiences of CSII.

**Table 2:** Selection criteria determined using the PICOS model

Selection criteria	Inclusion	Exclusion
Population	People with Type 1 or Type 2 diabetes People who have an insulin pump People who are considering using CSII Research from the perspective of health‐care professionals/carers/relatives	Non‐routine use of CSII (such as use specifically in pregnancy or in hospitals)
Intervention(s)	Routine use of CSII	No focus/data on experience of living with the pump Purely biomedical focus on the insulin pump Research focused on continuous glucose monitoring (CGM)
Comparison(s)	[none]	[none]
Outcome(s)	[none]	[none]
Study design(s)	Research protocols Qualitative Observational Methodological (including development work) Review Mixed methods	Purely quantitative
Publication type(s)	Peer‐reviewed original research article or review Databases and registers of on‐going studies	Patent Commentary Editorial
Publication year(s)	>2008	
Language(s)	English	

**Table 3:** Eligibility criteria for literature identified in the search

Inclusion	Studies examining some form of psychosocial aspect of living with CSII
	Peer‐reviewed original research or review
	Studies published from 2008 to March 2017
	Research using qualitative or mixed methods, as well as literature reviews, review papers, reports, conference papers
	Papers examining routine use of the pump
Exclusion	Abstracts that do not have a full‐text article available
	Papers not written in English
	Papers with a purely biomedical or quantitative focus

**Figure 1:**
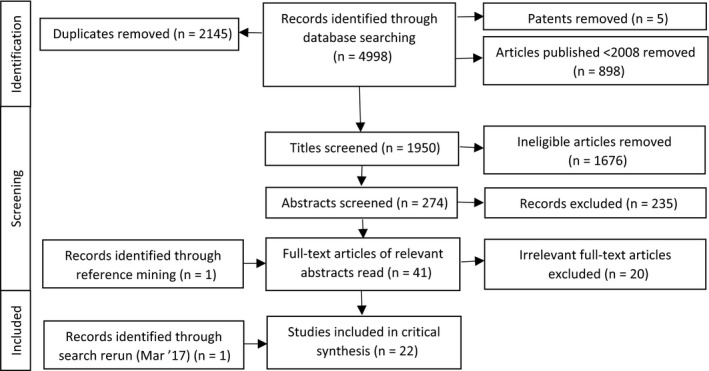
PRISMA flow diagram of identified articles

Duplicate papers were removed before screening (Figure 1). Titles and abstracts were screened by CR and a second reviewer from the team (split between MB, AR, AK and IV). Disagreements about inclusion were resolved at the title screen stage by third review (IV) and through discussion between CR, AK and AR at the abstract stage. Thirty‐nine full‐text articles were reviewed by both CR and AR, and one further article was identified through screening the reference lists of the full‐text articles. After exclusions, quality appraisal was performed by CR and AR using guidance from Dixon‐Woods et al.^101^ Included papers were deemed as mostly good quality based on this guidance, except for 2 which were included because of theoretical relevance.^59^ The final literature search was run in March 2017.

### 2.4 Data extraction and synthesis

Key information was extracted from papers using a data extraction form including (i) background information about each paper, (ii) key findings and themes identified by authors, (iii) references by authors in terms of implications and/or suggestions for improvement for incorporation of the device, (iv) critical interpretations by reviewers of key themes for CSII incorporation and (v) how/whether social support was defined/discussed. The data in the review constituted the main themes reported in each of the individual studies.^60^ Each paper was analysed in consideration of themes identified, after which the papers were systematically compared. CR reviewed full papers, and review findings were then discussed and refined with AR and IV in an iterative process. Where more than one paper contributed to a single theme, identifying numbers from the studies were noted at the end of each theme. This enabled relationships across the studies to be identified and provided the basis for a broader explanatory framework.

## 3 RESULTS

Twenty‐two studies were identified which described the experiences of CSII from the perspectives of children/adolescents/young adult pump users (9) (Participants n = 251), adult pump users (8) (Participants n = 143), HCPs (4) (Participants n = 61) and/or parents of pump users (7) (Participants n = 266). Eighteen of the papers were qualitative, and 4 used mixed methods. Contextual data from each of the papers are presented in Table 4.

**Table 4:** Contextual information about the included studies

Author(s), year, country	Aim	Study design	Perspective	Sample	Major findings
Wilson (2008), UK^68^	To gain the pump user's perspective of using a pump with the objective of exploring communicative process with HCPs + how and why people self‐manage their condition	Descrip, Tel‐ints	Pump user	N = 25, Age: 18‐80, Sex: 12M, 13F	If not pump‐trained, diabetes centres provided poor communication and lack of support for intensive diabetes self‐management. Some pump users did not attend these clinics, instead communicating with alternative sources for support and information. Individuals were motivated to continue CSII, despite barriers from HCPs
Everett et al (2010), UK^72^	To determine the barriers of achieving better glycaemic control	Descrip, FGs	Pump user	N = 17, Mean Age: 44 ± 13.3	Barriers were as follows: expectations of increased hypoglycaemia; anticipated restrictions to lifestyle; mistrust of HbA1c results; and the hard work associated with good glycaemic control. However, participants were eager to continue learning while HCPs need to learn from pump user experience. Hypo fear needs to be addressed early on in CSII pathway
Todres et al (2010), UK^78^	To provide in‐depth insight into the changes that may be experienced by people with diabetes embarking on CSII	Descrip, F2F ints	Pump user	N = 4, Age range: 21‐51, Sex: 2M, 2F	Switching from MDI to CSII provides challenges in the short term but over a longer period there are significant improvements in QoL for users. There is a change in the relationship between the pump user and HCP where successful implementation arises from a more collaborative relationship
Hayes et al (2011), UK^64^	To examine why people with T1D choose to discontinue CSII	Descrip, F2F ints	Pump user	N = 5, Sex: 2M, 3F	Main themes: the challenges of wearing the pump; the inconvenience of it; lack of control over the pump, body and health; and comparing expectations vs reality
Olinder et al (2001a), Sweden^67^	To gain insight into and generate theoretical knowledge about the processes involved when insulin pump‐treated adolescents take or miss taking their bolus doses	Descrip, F2F ints	Pump user, parent, HCP	N = 12, Age: 12‐19, Mean Age: 14.4, Sex: 5M, 7F + N = 4 parents + 1 DSN	“Lost focus” was identified as the main reason for missed bolus doses: forgetting to bolus post‐meal; distraction at mealtimes; the perceived impact of taking the bolus is too high (when around others/when fatigued with diabetes). Strategy involves agreements between adolescents and their parents about bolus reminders
Olinder et al (2011b), Sweden^77^	To discover the specific reasons why bolus doses are missed and what strategies exist to avoid this, from the adolescents’ point of view	Descrip, F2F ints	Pump user	N = 12, Age: 12‐19, Mean Age: 14.4, Sex: 5M, 7F	Responsibility in the context of taking or missing bolus doses emerged as the core category. There is a need to clarify the responsibility for SM in continuous negotiations between adolescents and parents to avoid missed doses. HCPs can facilitate and encourage these negotiations
Alsaleh et al (2012), (USA, UK, Sweden^11^	To identify studies that explore the experiences of children/young people and their parents on the transition from injections to insulin pump therapy, in the context of their daily life	Sys lit search	Pump user, parent	Various	Six studies identified. People with diabetes learned about CSII either formally from HCPs or informally from a friend/online. Advantages: improved diabetes control; a positive impact on the QoL from greater flexibility in lifestyles. Disadvantages: pump visibility; physical restrictions; day‐to‐day management. All participants preferred CSII to MDI, but there is a scarcity of psychosocial data; further research is needed
Alsaleh et al (2013), UK^73^	To determine the views and experiences of parents and children regarding the training and services they received at a London teaching hospital, when the child commenced insulin pump therapy, and to inform future services	Experi, F2F ints	Pump user, parent	N = 34, Age: 5‐17, Sex: 25M 17F, + N = 38 parents	The insulin pump therapy programme provided was appreciated by the majority of families, and provided children and their parents with support for easier transition from MDI to CSII
Garmo et al (2013), Sweden^61^	To describe experiences of the impact of insulin pump therapy in adults with T1D after >5 y use of an insulin pump	Descrip, F2F ints	Pump user	N = 16, Age: 29‐65, Median age: 55, Sex: 6M, 10F	The overarching theme revealed that insulin pump therapy was experienced as both a shackle and a lifeline. Six sub‐themes emerged: subjected vs empowered; dependent vs autonomous; vulnerable vs strengthened; routinized vs flexible; burdened vs relieved; and stigmatized vs normalized
Tullman (2013), USA^62^	To explore the individual experiences of female, adolescents with T1D wearing an insulin pump	Descrip, F2F ints	Pump user	N = 12, Age: 12‐28, Sex: 12F	Key positive themes: increased flexibility; increased perceived control over diabetes; higher level of self‐esteem. Key negative themes: increased awareness of own body; a constant struggle to maintain health; increased concern of body weight and relationship with food; lack of societal awareness of T1D and the pump. Also reports of general impact/change in intimate and peer relationships, although not necessarily negative
Alsaleh et al (2014), UK^52^	To examine the impact of switching from MDI to CSII on glycaemic control and daily lives of children/young people and their families	Descrip, F2F ints	Pump user, parent	N = 34, Age: 5‐17, Sex: 25M, 17F^a^ + N = 38 parents	Key positive themes: Significantly improved blood glucose values after 6 mo (8.2% vs 7.6%). Sustained over 3 y; CSII generally preferred over MDI; better general well‐being; feeling more in control of diabetes and live; more “normal” life. Parents described more healthy attitudes towards food; improved sleep patterns; more relaxed lifestyles; higher energy levels. Key negative theme: Most difficulty reported at the commencement of use
Barnard et al (2014), UK^75^	To explore the experiences of adolescents with T1D and their parents taking part in an overnight closed‐loop study at home, using qualitative and quantitative research methods	Experi, F2F ints	Pump user, parent	N = 15, Age: 12‐18, Mean age: 15.6 ± 2.1, Sex: 9M, 6F + N = 13 parents	Key positive themes: reassurance/peace of mind; confidence; “time off” from diabetes demands; safety; improved diabetes control. Key negative themes: difficulties with calibration, alarms, and size of the devices. Closed‐loop insulin delivery represents cutting‐edge technology in the treatment of T1DM. Results indicate that psychological and physical benefits outweighed practical challenges
Forsner et al (2014), Sweden^65^	To determine parents’ experiences of caring for a child <2 y old who had T1D and was being treated with CSII therapy	Descrip, Longit, F2F ints	Parent	N = 6 parents, Age: 25–40	Parents of infants with diabetes are in great need of support to manage the disease and CSII technology. The fear of losing control and the lack of relief lead to social isolation. Educating someone close to the family could be a valuable intervention
Saarinen et al (2014), Sweden^69^	To describe how people with T1D experience the transition from MDI to CSII	Descrip, FGs	Pump user	N = 11, Age: 25‐74, Mean age: 46, Sex: 6M, 5F	Key positive themes: Greater freedom and flexibility, particularly with meals; improved BG control. Those around users reacted with curiosity. Some pump users felt compelled to tell others that they had diabetes because the pump could be seen or heard. Coping with CSII in daily life was easier and more comfortable than expected. However, having to constantly be prepared for technical failure was cumbersome. Transition to CSII may be liberating, but also imply a sense of the diabetes made visible
Barnard et al (2015), UK^74^	To explore the psychosocial experiences of closed‐loop technology and to compare ratings of closed‐ and open‐loop technology for adults with T1D taking part in a randomized crossover study	Experi, F2F ints	Pump user	N = 24, Age: Mean 43 ± 12, Sex: 13M, 11F	Closed‐loop therapy can free participants from the demands of self‐management. Key negative themes: technical difficulties; “connectivity,” which it is hoped will improve. Key positive themes: improved blood glucose control; reassurance/reduced worry; improved overnight control leading to improved daily functioning and diabetes control; improved sleep. Key negative themes: technical difficulties; intrusiveness of alarms; size of equipment. Participants recommend closed‐loop technology
Hood and Duke (2015), USA^70^	To investigate the multidimensional meaning of living with an insulin pump while facing the challenges of life as an emerging adult	Experi, F2F ints	Pump user	N = 9, Age: 19‐24, Mean age: 20.9, Sex: 3M, 6F	T1D is like the process of learning tightrope walking; learning to live with diabetes unfolds over time and requires the walker to return to the wire after inevitable falls, trying to achieve a balance. Four themes represent the essence of the day‐to‐day experiences of these emerging adults: seeking control, becoming responsible, staying connected, and accepting me
O'Kane et al (2015), UK/Canada/USA^76^	To examine how T1D devices are adopted, carried, and used	Descrip, F2F ints, DS, GMU	Pump user	N = 41, Age: 23‐65, Sex: 10M, 31F	Negative themes: adoption of devices; carrying devices; use of devices in front of others. Difficulties Include interactions with; family, friends, colleagues, romantic partners, people while travelling, + strangers. Non‐routine events led to uncharacteristic hiding of diabetes/technology in uncertain social situations vs showing off the technology in social situations where there was something to gain. Wide variation in “Normal use” in familiar public situations such as routine work lives and personal lives. In public there is variation on whether pump users care what strangers think
Rankin et al (2015), UK^63^	To understand the impact on parents who care for young children using insulin pumps; to help interpret psychological outcomes reported in quantitative research; and to inform provision of support to future parents	Descrip, F2F ints	Parent	N = 19, Age: 34‐44, Mean age: 40.1 ± 3.7, Sex: 6M, 13F	Positive themes: no injections, fewer restrictions on child especially in relation to eating, better family life and glycaemic control. Negative themes: Additional and unanticipated work to manage their child's diabetes using a pump. Parents felt they would benefit from being made aware of the additional work involved, and also from education and support to address concerns. Better measures to evaluate parents’ concerns were also raised
Ferrari et al (2016), Australia^71^	To better understand the complexities of the lived experience of children and how this may differ across MDI and CSII treatment regimens	Descrip, Longit, F2F ints	Pump user	N = 17, Age: 7‐15, Mean age: 11.8 ± 2.4, Sex: 7M, 10F	Illness phase and treatment regimen shaped how bodily cues were interpreted. CSII allowed children to listen to and trust their bodily cues rather than override. Shame was a barrier to support engagement. Different internalized and externalized views of T1D emerged. Overall, children were insightful experts of their own experiences
Lawton et al (2016), UK^21^	To explore health professionals’ views about CSII and the types of individuals they thought would gain greatest clinical benefit from using this treatment	Descrip, F2F ints	HCP	N = 18, DSN: 12 Diet: 6, Prac: 5‐29	HCPs perceived CSII as offering better insulin therapy to some individuals. However, HCPs felt that CSII is more technically complex than MDI, and so, selected individuals based on whether potential users possessed attributes to enable optimal use of the technology. However HCPs assumptions had been challenged by working on the REPOSE trial by observing individuals making effective use of CSII who they would not have recommended
Shulman et al (2016), Canada^66^	To understand why pumps have been broadly adopted to inform optimal practice and the development of strategies to deal with pressures to adopt new technologies into practice	Descrip, F2F ints	HCP	N = 16, Sex: 8M, 8F, Phys: 16, Prac: 2.5‐45	Key themes: CSII may fall short of expectations of improved glycaemic control; although CSII deemed as limited in terms of this outcome, HCPs also saw where it had value both for the users and for themselves.### Pumps status as new technologies, which were seen to have current, or to promise future, benefits
Perry et al (2017), Australia^79^	To examine the support context for people with diabetes using CSII from the HCP perspective, as well as contextual influences for HCPs and people with diabetes	Descrip, Tel‐ints	HCP	N = 26, Sex, DNE: 12, Diet: 3, Phys: 8, GP: 3	Key themes: difficulties, disconnections, and disarray. Reports of shortages of HCP CSII expertise in practice + disconnected and disarrayed service structures and processes. Needs for consistent and coordinated care for people with CSII, and the infrastructure to facilitate this was highlighted

Descrip, Descriptive; Experi, Experimental; Sys lit, Systematic literature review; Longit, Longitudinal; Tel‐ints, Telephone interviews; F2F Ints, Face‐to‐face interviews; FGs, Focus groups; DS, Diary Study; GMU, Group meet‐up; M, Male; F, Female; DSN, Diabetes Specialist Nurse; DNE, Diabetes Nurse Educators; Diet, Dietician; Phys, Physician; GP, General Practitioner; Prac, Diabetes practice in years; No data, not provided in paper.

a Numbers do not add up.

From the data analysis, 3 themes of relevance emerged: “Tensions between expectations and experiences in adoption and early adaptation; Negotiating responsibility and accessing support from health‐care professionals and wider networks; and Reflexivity, active experimentation and feedback”.

### 3.1 Tensions between expectations and experiences in adoption and early adaptation

Polarization between expectations and experiences of users in learning to live with the technology was reported as common in the early stages of adoption. Prominent in the narratives was the device allowing for “increased flexibility” but accompanied by descriptions of on‐going disruption in daily activities, and needs for adjustment when initiating this type of insulin therapy.^61^, ^62^, ^63^

There were differences in people's initial expectations. Where some saw the pump as a panacea for insulin delivery, others simply saw the device as a tool, which incrementally improved existing efforts at diabetes‐related daily management by making subtle but useful adjustments.

In the way, simple. Really I think it is in the way and the fact that it didn't meet up to the expectations that I perhaps thought it would in overall control.

(Female who discontinued CSII)^64^

I have a very stationary job but I am fairly active at the weekends and then I can sometimes adjust to a temporary basal rate, or change the programme. I have a basal programme that is lower.

(Female, aged 52 years)^61^

This initial expectation (and potential contradiction) was seen as important to address by all parties (users/parents/HCPs)^21^, ^61^, ^62^, ^63^, ^64^, ^65^, ^66^ and perceptions of what the pump could do shaped subsequent expectations of the amount and nature of work required to master living with this new device.^63^, ^67^, ^68^ Reasons given by people with T1D for wanting to move to CSII from MDI included pursuit of greater stability and control over blood sugar levels, and desire for a more flexible lifestyle.^11^, ^69^, ^70^ Most users indicated that the new equipment made self‐management easier in terms of work required to balance glucose levels, enabling them greater flexibility in, for example; when/where/how they chose to eat; and undertaking spontaneous activities.^11^, ^21^, ^61^, ^62^, ^63^, ^65^, ^66^, ^67^, ^69^, ^71^

It just gets better and better; the transition from syringes to pump was painless. I think it is much easier to have the pump than all those syringes.

(Male, aged 25 years)^69^

By comparison, MDI was described by some as an insensitive approach to physiologically imitating insulin production, with inability to adjust levels of insulin in the body for up to 24 hours;

The pump allows me to obtain tight control of my blood glucose by administering very small amounts of insulin…which cannot be done with insulin injections…this prevents me having to have a higher basal rate…which leads to more hypos because it is too much insulin for me.

(Gender unknown, aged 25 years)^68^

However, improved self‐management and “flexibility” sat in tension with increased expectations to learn new “work”; new skills and adopt new practices associated with a more complex piece of technology.^11^, ^52^, ^61^, ^63^, ^64^, ^65^, ^69^, ^70^, ^72^, ^73^, ^74^ Parents reported “putting their life on hold”^52^ while integrating the new tool into management of their child's diabetes. Acquiring new skills relating to use and monitoring of the device was seen as tedious, challenging^11^, ^52^, ^63^ and burdensome with respect to the complexity and frequency of some tasks.^63^, ^74^

Considering where and how to wear this contraption on the body also illuminated constraints to “flexibility,” while a potential dissonance seemed to occur between expectations that insulin management is automatic and simplified with the reality of the new machine requiring substantial thought and action. Feelings of vulnerability were also described when there was an overwhelming need to prepare for potential failures in this new apparatus.^61^, ^64^, ^75^ This was often reported as illness‐burden, particularly in studies representing the views of adults and parents, and during the early stages of adoption.^52^, ^61^, ^64^, ^65^, ^69^, ^74^, ^75^

In addition to new work, the device also introduced new inflexibilities. Typical daily experiences of CSII were described in dichotomized terms as representing both a shackle and lifeline.^61^ For many, the pump was experienced as constraining because it interfered with day‐to‐day life, due to the increased visibility necessitating further work to discretely attach this piece of equipment to the body, and/or the status attributed to a permanent appendage to be worn 24/7.^52^, ^61^, ^62^, ^64^, ^65^, ^69^, ^70^, ^74^, ^76^ Physical restrictions were also imposed, which were caused by the bulky nature of the tool and its connecting tubes and alarms.

As a woman, I like tight skirts and dresses, I can't wear that anymore. I have to choose clothes based on the pump.

(Female 43 years)^69^

Women expressed more concern than men about body image and social acceptance in terms of the visibility and concealment of the pump.^64^, ^69^, ^76^ Parents were less concerned about practicalities of wearing the device, and more with the reliance and safety of the technology.^11^, ^52^, ^65^, ^73^, ^75^ These disadvantages illuminate discrepancies between expectations and realities of introducing a new health contraption, and a variety of experiences and perspectives on its adoption. Different demands in daily activities, and in user's preferences and priorities, mean that everyday contexts in which the device may be accommodated is variable, and that there are a range of “trade‐offs” between the limitations and advantages of CSII.

There were descriptions of persistent aloneness in trying to be “normal,” and trying to hide the equipment from others to achieve this. Many users of CSII expressed feelings of being different from peers, yet wanting to be and feel “normal”.^61^, ^62^, ^66^, ^70^, ^74^, ^76^, ^77^

When you take it out [the pump] you feel like you're exposing something about yourself for people to, sort of, either, sort of, judge that it's good or bad, in a way, and then I more, sort of, fear someone's reaction.

(Female, age unknown)^76^

Living with diabetes was described as a constant struggle, and the process of self‐management as isolating and lonely,^70^ especially when there was a potential for prying or judgement from others.^61^, ^62^, ^64^, ^69^, ^70^, ^71^, ^76^, ^77^, ^78^ Intimate relations were also discussed with reference to the inevitable awkwardness in explaining the device to a sexual partner, or the contraption getting in the way.^61^, ^62^, ^64^, ^69^, ^76^ While most users expressed being open to others, some preferred not to expose their diabetes diagnosis or means of insulin delivery to strangers.^61^, ^62^, ^64^, ^69^, ^70^, ^71^, ^76^, ^77^, ^78^ However, contemporary popular interest in innovation was thought to assist in explaining the condition to others, using the apparatus (a relatively familiar looking object) as opposed to injections (MDI).^52^, ^61^, ^62^, ^63^, ^69^, ^70^

### 3.2 Negotiating responsibility and accessing support from health‐care professionals and wider networks

This second theme highlights the ensuing need for emotional and practical assistance, and understanding of who is responsible for the management tasks relating to the pump, during the initial phase of adoption.

How individuals incorporate the device and undertake activities relies, to an extent, on external influences, encompassing a range of social and health‐care support‐related relationships. Users of CSII articulated how social support provided additional assistance, and how a network of support enabled the work of self‐management to be shared out.^62^, ^63^, ^67^, ^71^ HCPs also echoed the importance of on‐going multifaceted, holistic and tailored expertise in facilitating CSII use.^66^, ^79^ However, not all social interactions were viewed as beneficial to self‐management.

In the main, facilitation and encouragement from family members and HCPs in adapting to and understanding the mechanisms of the device were considered helpful by those living with this tool.^72^, ^78^ Mastering CSII, from the user's point of view, was described as easier when there was trust and assistance from HCPs, which was tailored and holistic.^61^, ^70^

I don't want my blood sugars to be high all the time or low all the time…But when [the HCP] adjusts stuff without looking at what's actually going on or listening, it's just kind of pointless.

(Sex unknown, young adult, exact age unknown)^70^

The complexity of the equipment could make users feel vulnerable in terms of needing backing to programme and manage its more advanced features.^61^, ^69^ Complex tasks included understanding how insulin is administered, and sharing practical tips for discrete/un‐invasive placement on the body (from other users or HCPs). Advocated assistance included provision of psychological support in clinics and play therapy for younger children. Aids to assist with subcutaneous cannula insertion, simplifying the process and easing pain, or testing a saline pump to experience how it feels to be attached to the device before implementation were also advised,^73^ as well as more information and interaction to set up the machine.^69^, ^71^, ^79^ However, too much information at initiation could be unhelpful. Not seeking or having any on‐going support or information about the equipment proved to be detrimental to incorporation.^64^, ^71^, ^79^

Assistance and information from others in a non‐clinical setting was identified as relevant. Insulin pump users described wanting to learn about the device and find ways to fit it into their lives through learning from peers (i.e people who actually have experience of living with T1D).^70^, ^72^

We're like, ‘How's your blood sugar?’… “We'll joke about it [blood sugar levels]. It's…reassuring, that other people are going through it too, you know. So you don't feel as weird…You feel kind of normal.

(Female, young adult, exact age unknown)^70^

Interactions with peers offered the prospect of support, shared learnings and practical solutions for day‐to‐day problems.^11^, ^62^, ^68^, ^70^, ^72^ Connecting with others through face‐to‐face contact or through blogs was considered valuable.^70^ Similarly, meeting other families was valued,^73^ where parents with some shared responsibility for managing this machine also reported on the initial burden.^11^, ^52^, ^65^ Valued elements of meeting others included; sharing the training experience, meeting others in the same situation and a relaxed atmosphere, which facilitated troubleshooting.

However, accessing aid, whether from peers, family members or HCPs was influenced by the level of responsibility taken, or desired, from the user.

It's starting to hit me now… I don't realize that the diabetes is damaging [my body] … It [having the pump] was just kind of a wake‐up call… is my responsibility … not my mom's.

(Female, young adult, exact age unknown)^70^

The level of responsibility is also seen to vary between age groups, from younger children needing more intensive parental assistance to adults wanting to feel a full sense of control over their diabetes, and incremental changes in desired responsibility in between.^61^, ^62^, ^64^, ^65^, ^67^, ^70^, ^72^, ^77^, ^78^

There are also times when the need for support varies (e.g in times of sickness). The establishment of distribution and transfer of responsibility from parents to children, adolescents and young adults was discussed. Parents often hold most of the responsibility of diabetes management for children, which is gradually handed over, to varying effect.^52^, ^62^, ^67^, ^70^, ^71^, ^73^, ^77^

But now, in the autumn she missed a little bit again [of insulin doses]. Then I realized that it's not possible to leave the responsibility to her so much, because it didn't work, she forgot doses and such.

(Mother)^67^

A hindrance to this transfer of responsibility could be parental fear of the ability of the child to self‐manage and so the parent may be reluctant to surrender responsibility. Parents were quoted as desiring education and assistance themselves.^65^, ^67^

### 3.3 Reflexivity, active experimentation and feedback

This theme focuses on challenges faced by new pump users, and the process of the integration and normalization of the device. The term “reflexivity” here refers to how experiences that interrupt what is normalized and/or habitual for individuals are encountered and understood consciously (i.e reflexively), and the implications that this has for how people then act and incorporate the new apparatus into their everyday lives.

Normalization of this piece of equipment as a new practice is a process of gradual acceptance and assimilation. Some respondents described how the device felt like a tattoo, an appendage, or an extension of self after the initial period of getting to grips with the new contraption, requiring a journey of reflection, active experimentation and feedback.^62^, ^65^, ^70^, ^76^

I was self‐conscious about [the pump] at first… I was like, ‘Ugh, people will see it’ [the pump] … But [going to diabetes camp] really got me out of my shell… It's like telling someone I got a new tattoo… It's [the pump] just a part of me.

(Male, young adult, exact age unknown)^70^

Adoption was predicted on a demand that the user trust the machine to perform its functions safely.^62^, ^64^, ^75^, ^78^ In addition to adjusting to the initial complexities, fear that the apparatus would do something that the user does not want it to, or not wanting to give up control suggests psychological adjustments alongside other practical adjustments.

At night I can't help think that if the buttons pressed or … even in the day if you knock it [the pump] or something goes in or too much, you haven't got full control over what you are putting in your body really so that was part of it as well

(Female who discontinued CSII)^64^

Over time, the initial stress and vulnerability created by dependence on a machine gave way to feelings of autonomy when the technology was mastered.^61^, ^64^, ^65^, ^66^, ^69^, ^70^, ^73^, ^74^, ^75^

You have to be a bit knowledgeable as well and you have to learn about the pump yourself very carefully… It's a case of having the courage to try the different functions of the pump, so you know what to do if something goes wrong.

(Female, aged 54 years)^61^

Users of CSII reported the need for a period of adjustment to feel comfortable with being attached to a machine 24 hours a day.^61^, ^62^, ^64^, ^67^, ^69^, ^70^, ^72^, ^76^, ^77^, ^78^

The visibility of the device created a sense of heightened awareness of one's body and as a result a greater need for assistance to adapt and find ways to comfortably situate the machine at the point of introduction.^52^, ^62^, ^69^, ^73^ Through technical control of the apparatus, and resulting stabilized blood glucose levels, greater personal control was realized.^69^, ^78^ A common depiction of incorporation involved the need to gain motivation and confidence to adapt it.^21^, ^52^, ^61^, ^65^, ^68^, ^69^, ^70^, ^78^ For example, a parent of a young child using CSII commented on how longer term benefits were predicated on performing necessary work during the adoption phase;

You take care of it [diabetes] yourself. It's freedom with responsibility. That demands courage.

(Parent)^65^

## 4 DISCUSSION

This review suggests a period of adjustment and experience that emerges over time, and a process of incorporation that changes from the point of anticipation (pre‐CSII) through to adoption. This process is accompanied by having to navigate and be responsive to a range of contingent bodily sensations and technological demands that were unexpected at the outset. There is an initial liminality associated with use of the pump as a foreign object, and upon introduction users feel that they are on the edge of something new. People living with diabetes who adopt CSII do so with existing experiential knowledge of their condition; as such the process of adjustment necessary to embed this technology into everyday life includes integration of new knowledge about management combined with their existing understandings.

Initial expectations shape both the type and amount of work the person subsequently puts in to adopting and integrating the device into his/her daily life. Negotiation of responsibility and access to personalized information, support and resources can affect how well s/he is able to incorporate CSII. What follows is a need to engage in active experimentation, in which the user reflects on his/her experience and feeds that back into use of the appliance, adapting it to his/her needs. This can also be facilitated through negotiation of the assistance available to him/her (e.g shared experiences of other users of CSII, feedback from HCPs). The more the new pump user becomes accustomed to the tool, its physical presence and the greater the degree of aid available to him/herself and his/her families/significant others, the easier it can be incorporated.

This review suggests a qualitative difference between using MDI and CSII which centres on experiencing metabolic improvements, but also to feelings of ease, personal control and confidence in using and habituating to more complex technology. The apparatus evokes feelings of technological advancement and flexibility, and so high expectations of the device's potential are engendered. The previous method of insulin delivery required needles, a very physical but singular interaction, whereas this machine is integrated into the body 24/7. This process can make users much more aware of their body image and appearance. Additionally, using CSII introduces new types of work, the completion and normalization of which requires acquiring new skills and renegotiating relations within personal communities.

The review also suggests that if a new user of CSII has no access to additional support or resources, then their ability to incorporate the new appliance will be hindered. It has been found that effective diabetes medical care and self‐management is enriched by improving access to specialist and on‐going diabetes HCPs.^80^, ^81^, ^82^ However, HCPs providing care for patients with diabetes do not currently receive postgraduate training for the relief or assessment of educational, medical, emotional or psychological aspects of diabetes.^83^ Other means to supplement this support are therefore vital. Many aspects of self‐management are more achievable through working with others, by allowing knowledge, skills and resources to be pooled.^84^, ^85^

The, very recent (post‐March 2017), Relative Effectiveness of Pumps Over MDI and Structured Education (REPOSE) trial,^86^ compared CSII with MDI, with findings that resonate with this current review including pump expectations not being met but experiencing; increased discretion, flexibility and spontaneity (especially with food or exercise). The report, however, focused on improvements in diabetes self‐management due to structured education and on‐going support. Studies considered here indicate that there is a potentially stressful element in introducing a new and complex technology into someone's life. The role of others in accessing assistance could be a future avenue to explore. What we do know is that social networks and good social support are associated with better functioning, fewer psychosocial problems and improved self‐management in people with diabetes in general.^87^, ^88^ Social networks can provide emotional and/or practical aid as well as facilitating a means to mobilize, negotiate, mediate and access further means of assistance.^89^, ^90^, ^91^, ^92^ A supportive social network is known to have a “buffering” effect in situations eliciting stress (such as the introduction of a complex new technology),^93^, ^94^ but the impact of social networks amongst people living with CSII is not well, or reliably, documented.^95^ When CSII is first introduced, the level of responsibility taken for pump management is as much as the user is willing to accept, and this varies. The desire for responsibility of self‐management is thought to increase from childhood through to adulthood, and negotiation with caregivers is required to share out tasks. The findings in this synthesis not only resonate with and compliment research on social networks in long‐term conditions (outlined above), but also with studies examining shared responsibility between adolescents with T1D and their caregivers.^96^, ^97^ While motivation to take responsibility for self‐management is important,^81^, ^98^ motivation is not all that is required, as people living with T1D may, for example, feel fatigued. Sharing responsibility for the work of managing the condition can enable better self‐management and improved health outcomes through sharing the illness and CSII‐related burden associated with the complexity, frequency and relentless nature of some self‐management tasks.^98^, ^99^ This is where a link to support and resources could prove crucial.

### 4.1 Implications

These findings identify the types of beliefs that influence the adoption and diffusion of technologies. In terms of CSII, barriers to incorporation for the person with diabetes include the tension between the expectations of the device and the actual experience. For improved integration, early conversations are needed from HCPs about the likely period of disruption. Potential pump users have not been familiarized with the work that is going to be carried out, and they need time, resources and information to overcome this. HCPs and manufacturers of CSII need to be realistic with potential users so that they can anticipate this work. Frank conversations about the limitations of the apparatus are necessary. People with diabetes need to be given the opportunity to build confidence about using this new appliance, and negotiations between children/adolescents and their parents must be undertaken. Being prepared for the time required to work the contraption into their lives, as well as sensitivity to the inevitable variability between users could set realistic expectations. Harrison^100^ described how perceived assistance from HCPs or peers formed an important aspect of patient satisfaction and should be considered for future interventions. In examining the social network that pump users have access to, and enabling them to tap into further (and on‐going) means of support and resources, users of CSII could incorporate the apparatus more successfully.

### 4.2 Limitations

A number of limitations must be acknowledged with respect to the present review. Firstly, the findings of the synthesis reflect the background and experiences of the reviewers, and as such are subjective. We acknowledge that the findings could have been different if conducted by a different set of researchers, however, steps have been taken in line with guidance^101^ to ensure transparency in reporting on analytic processes which informed our analyses. Secondly, the papers included in the review incorporated a variety of methods, meaning that data quality was variable. The authors were sensitive to the quality of the methodology and did bear this in mind throughout the data analysis, and no concerns were raised with respect to the veracity of reporting or integrity of findings.

Thirdly, while men and women were, roughly, equally represented as participants in the papers reviewed (where these were reported; 44% vs 56%), it appears that men were relatively underrepresented in the quotes given in the papers (15% vs 45%—with the remaining 40% of quotes being non‐gender specific). Therefore, quotes offered in this synthesis of papers could potentially offer a pump adoption experience that is skewed towards female users. One possible contributor to this gender imbalance could be that more women expressed fears and concerns relating to body image and social acceptance than male participants.

Fourthly, reporting on demographic composition of study samples was not consistent across the papers reviewed. For example, not all studies disclosed the mean/median age^21^, ^62^, ^64^, ^65^, ^66^, ^68^, ^76^, ^78^, ^79^ or range^64^, ^72^, ^74^ of their participants. For those that did, the range was from 5 to 80, and of HCPs, the range of years in practice was 2.5‐45. The papers included a range of ages (children, adolescents, young adults, adults) and perspective (users of CSII, parents, HCPs), which offered an array of insights. However, saturation was not reached for any demographic group or perspective. Future studies may therefore look to explore comparatively the experiences of subgroups within the population of CSII adoptees and their families/significant others.

## 5 CONCLUSION

This review makes several original contributions to the knowledge base relating to experiences of pump users adoption and use; (i) investigation of recent studies not included in previous reviews of CSII device adoption; (ii) synthesis of lived experiences of users of various ages, in greater depth; (iii) synthesis of perspectives from parents and HCPs. To our knowledge, this review also represents the first to explore, qualitatively and pragmatically, the process of incorporating a new technology, worn 24/7, in a long‐term condition.

## CONFLICT OF INTERESTS

None.

## References

[1] Diabetes UK (2016) . Facts and Stats. [online] London: Diabetes UK, pp.1‐17. Available at: https://diabetes-resources-production.s3-eu-west-1.amazonaws.com/diabetes-storage/migration/pdf/DiabetesUK_Facts_Stats_Oct16.pdf [Accessed 13 Dec. 2018].

[2] Hoogma RP , Hammond PJ , Gomis R , et al. Comparison of the effects of continuous subcutaneous insulin infusion (CSII) and NPH‐based multiple daily insulin injections (MDI) on glycaemic control and quality of life: results of the 5‐nations trial. Diabet Med. 2006;23:141‐147. 16433711 10.1111/j.1464-5491.2005.01738.x

[3] Hoogma RPL , Hoekstra JB , Michels BP , Levi M . Comparison between multiple daily insulin injection therapy (MDI) and continuous subcutaneous insulin infusion therapy (CSII), results of the five nations study. Diabetes Res Clin Pract. 2006;74:S144‐S147.

[4] Kerr D , Nicholls H , James J . Continuous subcutaneous insulin infusion (CSII insulin pump therapy) for type 1 diabetes: a Bournemouth perspective. Pract Diabetes Int. 2008;25:114‐117.

[5] Hilliard ME , Goeke‐Morey M , Cogen FR , Henderson C , Streisand R . Predictors of diabetes‐related quality of life after transitioning to the insulin pump. J Pediatr Psychol. 2009;34:137‐146. 18577542 10.1093/jpepsy/jsn062 PMC2722121

[6] Muller‐Godeffroy E , Treichel S , Wagner VM . Investigation of quality of life and family burden issues during insulin pump therapy in children with Type 1 diabetes mellitus–a large‐scale multicentre pilot study. Diabet Med. 2009;26:493‐501. 19646189 10.1111/j.1464-5491.2009.02707.x

[7] Lynch C , Qazi S , Stalker AJ , et al. Insulin pump (CSII) therapy improves the Quality of Life (QoL) in patients with diabetes: the derby experience. Diabet Med. 2010;27(Suppl 1):153.

[8] Misso Marie L , Egberts Kristine J , Page M , O'Connor D , Shaw J . Continuous subcutaneous insulin infusion (CSII) versus multiple insulin injections for type 1 diabetes mellitus. Cochrane Database Syst Rev. 2010;(1):CD005103. 20091571 10.1002/14651858.CD005103.pub2 PMC12582037

[9] Yi‐Frazier JP , Smith RE , Vitaliano PP , et al. A person‐focused analysis of resilience resources and coping in diabetes patients. Stress Health. 2010;26:51‐60. 20526415 10.1002/smi.1258 PMC2880488

[10] Clark LF , Bilbie JC , Abraham P . What do patients prefer: insulin pumps or multiple daily injections and structured education? A retrospective audit and patient questionnaire. Pract Diabetes Int. 2011;28:73‐75.

[11] Alsaleh FM , Smith FJ , Taylor KM . Experiences of children/young people and their parents, using insulin pump therapy for the management of type 1 diabetes: qualitative review. J Clin Pharm Ther. 2012;37:140‐147. 21729118 10.1111/j.1365-2710.2011.01283.x

[12] Cropper J , Kanchi L , Ford‐Adams M , Hulse T , Buchanan C , Barker E . Quality of life and HbA1c outcomes in children and young people commencing insulin pump therapy. Endocrine Abstracts. 2012;30:18‐19.

[13] Salehi S , Addington H , Turner K , Vithian K . An audit investigating how clinical outcomes on pump services can be achieved through intensive follow‐up via face‐to‐face and distance consultation. Diabet Med. 2014;31(Suppl 1):74.

[14] Bonfanti R , Lepore G , Bozzetto L , et al. Survey on the use of insulin pumps in Italy: comparison between pediatric and adult age groups (IMITA study). Acta Diabetol. 2016;53:403‐412. 26429560 10.1007/s00592-015-0810-4

[15] Ghazanfar H , Rizvi SW , Khurram A , Orooj F , Qaiser I . Impact of insulin pump on quality of life of diabetic patients. Indian J Endocrinol Metab. 2016;20:506‐511. 27366717 10.4103/2230-8210.183472 PMC4911840

[16] Barnard KD , Skinner TC . Qualitative study into quality of life issues surrounding insulin pump use in type 1 diabetes. Pract Diabetes Int. 2007;24:143‐148.

[17] Bode BW , Steed RD , Davidson PC . Reduction in severe hypoglycemia with long‐term continuous subcutaneous insulin infusion in type I diabetes. Diabetes Care. 1996;19:324‐327. 8729154 10.2337/diacare.19.4.324

[18] McMahon SK , Airey FL , Marangou DA , et al. Insulin pump therapy in children and adolescents: improvements in key parameters of diabetes management including quality of life. Diabet Med. 2005;22:92‐96. 15606698 10.1111/j.1464-5491.2004.01359.x

[19] Silverstein J , Klingensmith G , Copeland K , et al. Care of children and adolescents with type 1 diabetes. A statement of the American Diabetes Association. Diabetes Care. 2005;28:186‐212. 15616254 10.2337/diacare.28.1.186

[20] Kesavadev J , Das AK , Unnikrishnan R , et al. Use of insulin pumps in India: suggested guidelines based on experience and cultural differences. Diabetes Technol Ther. 2010;12:823‐831. 20807118 10.1089/dia.2010.0027 PMC2956384

[21] Lawton J , Kirkham J , Rankin D , et al. Who gains clinical benefit from using insulin pump therapy? A qualitative study of the perceptions and views of health professionals involved in the Relative Effectiveness of Pumps over MDI and Structured Education (REPOSE) trial. Diabet Med. 2016;33:243‐251. 26248590 10.1111/dme.12879

[22] Weissberg‐Benchell J , Antisdel‐Lomaglio J , Seshadri R . Insulin pump therapy: a meta‐analysis. Diabetes Care. 2003;26:1079‐1087. 12663577 10.2337/diacare.26.4.1079

[23] Bruttomesso D , Crazzolara D , Maran A , et al. In Type 1 diabetic patients with good glycaemic control, blood glucose variability is lower during continuous subcutaneous insulin infusion than during multiple daily injections with insulin glargine. Diabet Med. 2008;25:326‐332. 18307459 10.1111/j.1464-5491.2007.02365.x

[24] Pickup JC , Sutton AJ . Severe hypoglycaemia and glycaemic control in Type 1 diabetes: meta‐analysis of multiple daily insulin injections compared with continuous subcutaneous insulin infusion. Diabet Med. 2008;25:765‐774. 18644063 10.1111/j.1464-5491.2008.02486.x

[25] Riveline JP , Jollois FX , Messaoudi N , et al. Insulin‐pump use in everyday practice: data from an exhaustive regional registry in France. Diabetes Metab. 2008;34:132‐139. 18289909 10.1016/j.diabet.2007.10.010

[26] Chellamuthu P , Lawrence IG , Kitchener D , et al. Continuous subcutaneous insulin infusion: experience from a large specialist service: are we following NICE guidance and what is the added benefit of the DAFNE programme? Pract Diabetes Int. 2009;26:60‐64.

[27] Pankowska E , Blazik M , Dziechciarz P , Szypowska A , Szajewska H . Continuous subcutaneous insulin infusion vs. multiple daily injections in children with type 1 diabetes: a systematic review and meta‐analysis of randomized control trials. Pediatr Diabetes. 2009;10:52‐58. 18761648 10.1111/j.1399-5448.2008.00440.x

[28] de Bock M , Gunn AJ , Holt JA , et al. Impact of insulin pumps on glycaemic control in a pump‐naive paediatric regional population. J Paediatr Child Health. 2012;48:247‐252. 22085335 10.1111/j.1440-1754.2011.02245.x

[29] Pickup JC . Management of diabetes mellitus: is the pump mightier than the pen? Nat Rev Endocrinol. 2012;8:425‐433. 22371161 10.1038/nrendo.2012.28

[30] Shanmugasundaram M , Karamat M , Hand J , Field A , Charlton M , Dyer P . The impact of continuous subcutaneous insulin infusion in patients with Type 1 diabetes. Diabet Med. 2012;29(Suppl 1):175.

[31] Johnson SR , Cooper MN , Jones TW , Davis EA . Long‐term outcome of insulin pump therapy in children with type 1 diabetes assessed in a large population‐based case‐control study. Diabetologia. 2013;56:2392‐2400. 23963323 10.1007/s00125-013-3007-9

[32] Quiros C , Gimenez M , Rios P , et al. Long‐term outcome of insulin pump therapy: reduction of hypoglycaemia and impact on glycaemic control. Diabet Med. 2016;33:1422‐1426. 26870914 10.1111/dme.13094

[33] Alcolado J , Bailey K , Cuthbert S , Warren S . CSII therapy: the Cardiff experience. Diabetes Prim Care. 2008;10:103‐106.

[34] Jakisch BI , Wagner VM , Heidtmann B , et al. Comparison of continuous subcutaneous insulin infusion (CSII) and multiple daily injections (MDI) in paediatric Type 1 diabetes: a multicentre matched‐pair cohort analysis over 3 years. Diabet Med. 2008;25:80‐85. 18199134 10.1111/j.1464-5491.2007.02311.x

[35] Dissanayake S , Sittampalam V , Kong N . Audit on continuous subcutaneous insulin infusion (insulin pump) therapy in a district general hospital. Diabet Med. 2009;24(Suppl 1):81.

[36] Gane J , White B , Christie D , Viner R . Systematic review and meta‐analysis of insulin pump therapy in children and adolescents with type 1 diabetes. Arch Dis Child. 2010;95(Suppl 1):A94.

[37] Cook CB , Beer KA , Seifert KM , Boyle ME , Mackey PA , Castro JC . Transitioning insulin pump therapy from the outpatient to the inpatient setting: a review of 6 years’ experience with 253 cases. J Diabetes Sci Technol. 2012;6:995‐1002. 23063024 10.1177/193229681200600502 PMC3570832

[38] National Institute for Health and Clinical Excellence . NICE Technology Appraisal Guidance 151. Continuous Subcutaneous Insulin Infusion for the Treatment of Diabetes Mellitus (Review of Technology Appraisal Guidance 57). London: National Institute for Health and Clinical Excellence; 2008.

[39] Nicolucci A , Maione A , Franciosi M , et al. Quality of life and treatment satisfaction in adults with Type 1 diabetes: a comparison between continuous subcutaneous insulin infusion and multiple daily injections. Diabet Med. 2008;25:213‐220. 18201210 10.1111/j.1464-5491.2007.02346.x

[40] Carreira M , Anarte MT , Colomo N , et al. Effect of a telemedicine system on adherence to treatment in patients with type 1 diabetes and insulin‐pump therapy. Diabetes Technol Ther. 2013;15:A102.

[41] Pickup J , Mattock M , Kerry S . Glycaemic control with continuous subcutaneous insulin infusion compared with intensive insulin injections in patients with type 1 diabetes: meta‐analysis of randomised controlled trials. BMJ. 2002;324:705. 11909787 10.1136/bmj.324.7339.705 PMC99054

[42] Aathira R , Jain V . Advances in management of type 1 diabetes mellitus. World J Diabetes. 2014;5:689‐696. 25317246 10.4239/wjd.v5.i5.689 PMC4138592

[43] Johnson SB , Kelly M , Henretta JC , Cunningham WR , Tomer A , Silverstein JH . A longitudinal analysis of adherence and health status in childhood diabetes. J Pediatr Psychol. 1992;17:537‐553. 1432480 10.1093/jpepsy/17.5.537

[44] Kitabchi AE , Umpierrez GE , Miles JM , Fisher JN . Hyperglycemic crises in adult patients with diabetes. Diabetes Care. 2009;32:1335‐1343. 19564476 10.2337/dc09-9032 PMC2699725

[45] The Diabetes Control and Complications Trial Research Group . The effect of intensive treatment of diabetes on the development and progression of long‐term complications in insulin‐dependent diabetes mellitus. N Engl J Med. 1993;329:977‐986. 8366922 10.1056/NEJM199309303291401

[46] Wilmot EG , Choudhary P , Grant P , Hammond P . Insulin pump therapy: a practical guide to optimising glycaemic control. Pract Diabetes. 2014;31:121‐125a.

[47] Pickup J , Keen H . Continuous subcutaneous insulin infusion at 25 years: evidence base for the expanding use of insulin pump therapy in type 1 diabetes. Diabetes Care. 2002;25:593‐598. 11874953 10.2337/diacare.25.3.593

[48] American Diabetes Association . Standards of medical care in diabetes 2017. Diabetes Care. 2017;40(Suppl 1):S4‐S5. 28637892 10.2337/dc17-0299

[49] Mecklenburg RS , Benson JW Jr , Becker NM , et al. Clinical use of the insulin infusion pump in 100 patients with type I diabetes. N Engl J Med. 1982;307:513‐518. 7099222 10.1056/NEJM198208263070901

[50] McAdams BH , Rizvi AA . An overview of insulin pumps and glucose sensors for the generalist. J Clin Med. 2016;5:5. 10.3390/jcm5010005 PMC4730130 26742082

[51] Low KG , Massa L , Lehman D , Olshan JS . Insulin pump use in young adolescents with type 1 diabetes: a descriptive study. Pediatr Diabetes. 2005;6:22‐31. 15787898 10.1111/j.1399-543X.2005.00089.x

[52] Alsaleh FM , Smith FJ , Thompson R , Al‐Saleh MA , Taylor KMG . Insulin pump therapy: impact on the lives of children/young people with diabetes mellitus and their parents. Int J Clin Pharm. 2014;36:1023‐1030. 25108411 10.1007/s11096-014-9990-1

[53] Steineck I , Cederholm J , Eliasson B , et al. Insulin pump therapy, multiple daily injections, and cardiovascular mortality in 18 168 people with type 1 diabetes: observational study. BMJ. 2015;350:h3234. 26100640 10.1136/bmj.h3234 PMC4476263

[54] Hussain T , Akle M , Nagelkerke N , Deeb A . Comparative study on treatment satisfaction and health perception in children and adolescents with type 1 diabetes mellitus on multiple daily injection of insulin, insulin pump and sensor‐augmented pump therapy. SAGE Open Med. 2017;5:2050312117694938. 28321303 10.1177/2050312117694938 PMC5347412

[55] Pickup JC . Point: are insulin pumps underutilized in type 1 diabetes? Yes. Diabetes Care. 2006;29:1449‐1452. 16732043 10.2337/dc06-0011

[56] White HD , Goenka N , Furlong NJ , et al. The U.K. service level audit of insulin pump therapy in adults. Diabetic medicine. 2014;31:412‐418. 24117515 10.1111/dme.12325

[57] Barnard K. D. , Lloyd C. E. , and Skinner T. C. Systematic literature review: quality of life associated with insulin pump use in Type 1 diabetes. Diabetic Medicine. 2007;24:607‐617. doi:10.1111/j.1464-5491.2007.02120.x 17367304

[58] Noblit G , Hare R . Meta‐ethnography: Synthesising Qualitative Studies. Newbury Park, CA: Sage; 1988.

[59] Moher D , Liberati A , Tetzlaff J , Altman DG . Preferred reporting items for systematic reviews and meta‐analyses: the PRISMA statement. BMJ. 2009;339:b2535. 19622551 10.1136/bmj.b2535 PMC2714657

[60] Thomas J , Harden A . Methods for the thematic synthesis of qualitative research in systematic reviews. BMC Med Res Methodol. 2008;8:45. 18616818 10.1186/1471-2288-8-45 PMC2478656

[61] Garmo A , Hörnsten Å , Leksell J . ‘The pump was a saviour for me’. Patients’ experiences of insulin pump therapy. Diabet Med. 2013;30:717‐723. 23398606 10.1111/dme.12155

[62] Tullman AJ . A Phenomenological Study of the Psychosocial Effects of Insulin Pump Therapy on the Body Image and Self‐esteem of Female, Adolescent, Insulin Dependent Diabetics. Chicago, IL: The Chicago School of Professional Psychology; 2013.

[63] Rankin D , Harden J , Noyes K , Waugh N , Barnard K , Lawton J . Parents’ experiences of managing their child's diabetes using an insulin pump: a qualitative study. Diabet Med. 2015;32:627‐634. 25581347 10.1111/dme.12683

[64] Hayes M , Frearson S , Keller C , Cartmale A , Lewis‐Hayes S . A hermeneutic phenomenological study of why adults with type 1 diabetes choose to discontinue CSII. Euro Diabetes Nurs. 2011;8:12‐16.

[65] Forsner M , Berggren J , Masaba J , Ekbladh A , Olinder AL . Parents’ experiences of caring for a child younger than two years of age treated with continuous subcutaneous insulin infusion. Euro Diabetes Nurs. 2014;11:7‐12.

[66] Shulman R , Miller FA , Daneman D , Guttmann A . Valuing technology: a qualitative interview study with physicians about insulin pump therapy for children with type 1 diabetes. Health Policy. 2016;120:64‐71. 26563632 10.1016/j.healthpol.2015.10.006

[67] Olinder AL , Kerstin Ternulf N , Bibbi S . Clarifying responsibility for self‐management of diabetes in adolescents using insulin pumps‐a qualitative study. J Adv Nurs. 2011;67:1547‐1557. 21323979 10.1111/j.1365-2648.2010.05588.x

[68] Wilson V . Barriers to effective communication between patients using insulin pump therapy technology to enable intensive diabetes self‐management and the health professionals providing their diabetes care. J Assist Technol. 2008;2:26‐33.

[69] Saarinen T , Fernström L , Brorsson A‐L , Olinder AL . Insulin pump therapy is perceived as liberating, but to many it can imply a sense of the diabetes made visible. Euro Diabetes Nurs. 2014;11:38‐42.

[70] Hood DG , Duke G . The nature and meaning of insulin pump use in emerging adults with type 1 diabetes. Diabetes Spectr. 2015;28:75‐81. 25987805 10.2337/diaspect.28.2.75 PMC4433076

[71] Ferrari M , McIlwain DJ , Ambler G . A qualitative comparison of needles and insulin pump use in children with type 1 diabetes. J Health Psychol. 2016;21 http://journals.sagepub.com/doi/citedby/10.1177/1359105316653999. 10.1177/1359105316653999 27338629

[72] Everett J , Bowes A , Kerr D . Barriers to improving glycaemic control in CSII. J Diabetes Nurs. 2010;14:176‐181.

[73] Alsaleh FM , Smith FJ , Thompson R , Taylor KMG . A structured educational insulin pump therapy programme: the views of children/young people and their parents. Euro Diabetes Nurs. 2013;10:25‐30.

[74] Barnard KD , Wysocki T , Thabit H , et al. Psychosocial aspects of closed‐ and open‐loop insulin delivery: closing the loop in adults with Type 1 diabetes in the home setting. Diabet Med. 2015;32:601‐608. 25615888 10.1111/dme.12706

[75] Barnard KD , Wysocki T , Allen JM , et al. Closing the loop overnight at home setting: psychosocial impact for adolescents with type 1 diabetes and their parents. BMJ Open Diabetes Res Care. 2014;2:e000025. 10.1136/bmjdrc-2014-000025 PMC4212573 25452866

[76] O'Kane A , Rogers Y , Blandford A . Concealing or revealing mobile medical devices? Designing for onstage and offstage presentation. Proceedings of the 2015 Conference on Human Factors in Computing Systems (CHI‐2015); April 18–23, 2015, 2015; Seoul, Republic of Korea.

[77] Olinder AL , Nyhlin KT , Smide B . Reasons for missed meal‐time insulin boluses from the perspective of adolescents using insulin pumps: ‘lost focus’. Pediatr Diabetes. 2011;12(4pt2):402‐409. 21129137 10.1111/j.1399-5448.2010.00688.x

[78] Todres L , Keen S , Kerr D . Continuous subcutaneous insulin infusion in Type 1 diabetes: patient experiences of ‘living with a machine’. Diabet Med. 2010;27:1201‐1204. 20873363 10.1111/j.1464-5491.2010.03058.x

[79] Perry L , James S , Gallagher R , Dunbabin J , Steinbeck K , Lowe J . Supporting patients with type 1 diabetes using continuous subcutaneous insulin infusion therapy: difficulties, disconnections, and disarray. J Eval Clin Pract. 2017;23:719‐724. 28220558 10.1111/jep.12703

[80] Diabetes UK . State of the Nation England: Challenges for 2015 and Beyond. London: Diabetes UK; 2015.

[81] Casey D , Murphy K , Lawton J , White FF , Dineen S . A longitudinal qualitative study examining the factors impacting on the ability of persons with T1DM to assimilate the Dose Adjustment for Normal Eating (DAFNE) principles into daily living and how these factors change over time. BMC Public Health. 2011;11:672. 21878104 10.1186/1471-2458-11-672 PMC3175192

[82] Funnell MM , Brown TL , Childs BP , et al. National standards for diabetes self‐management education. Diabetes Care. 2008;31(Suppl 1):S97‐S104. 18165344 10.2337/dc10-S097 PMC2797381

[83] Byrne JL , Davies MJ , Willaing I , et al. Deficiencies in postgraduate training for healthcare professionals who provide diabetes education and support: results from the Diabetes Attitudes, Wishes and Needs (DAWN2) study. Diabet Med. 2017;4:1074‐1083. 10.1111/dme.13334 28195662

[84] Bandura A . Exercise of human agency through collective efficacy. Curr Dir Psychol Sci. 2000;9:75‐78.

[85] Bandura A . Health promotion from the perspective of social cognitive theory. Psychol Health. 1998;13:623‐649.

[86] Heller S , White D , Lee E , et al. A cluster randomised trial, cost‐effectiveness analysis and psychosocial evaluation of insulin pump therapy compared with multiple injections during flexible intensive insulin therapy for type 1 diabetes: the REPOSE Trial. Health Technol Assess. 2017;21:1‐278. 10.3310/hta21200 PMC5421095 28440211

[87] Kyngas H . Compliance of adolescents with diabetes. J Pediatr Nurs. 2000;15:260‐267. 10969500 10.1053/jpdn.2000.6169

[88] Karlsson A , Arman M , Wikblad K . Teenagers with type 1 diabetes – a phenomenological study of the transition towards autonomy in self‐management. Int J Nurs Stud. 2008;45:562‐570. 17046768 10.1016/j.ijnurstu.2006.08.022

[89] Kennedy A , Vassilev I , James E , Rogers A . Implementing a social network intervention designed to enhance and diversify support for people with long‐term conditions. A qualitative study. Implement Sci. 2016;11:27. 26926837 10.1186/s13012-016-0384-8 PMC4772323

[90] Hempler NF , Joensen LE , Willaing I . Relationship between social network, social support and health behaviour in people with type 1 and type 2 diabetes: cross‐sectional studies. BMC Public Health. 2016;16:198. 26926867 10.1186/s12889-016-2819-1 PMC4772283

[91] Vassilev I , Rogers A , Sanders C , et al. Social networks, social capital and chronic illness self‐management: a realist review. Chronic Illn. 2011;7:60‐86. 20921033 10.1177/1742395310383338

[92] Blickem C , Kennedy A , Vassilev I , Morris R , Brooks H , Jariwala P . Linking people with long‐term health conditions to healthy community activities: development of patient‐Led assessment for network support (PLANS). Health Expect. 2013;16:e48‐e59. 23731452 10.1111/hex.12088 PMC3908360

[93] Cohen S , Wills TA . Stress, social support, and the buffering hypothesis. Psychol Bull. 1985;98:310‐357. 3901065

[94] Miller TA , DiMatteo MR . Importance of family/social support and impact on adherence to diabetic therapy. Diabetes Metab Syndr Obes. 2013;6:421‐426. 24232691 10.2147/DMSO.S36368 PMC3825688

[95] Ritholz MD , Smaldone A , Lee J , Castillo A , Wolpert H , Weinger K . Perceptions of psychosocial factors and the insulin pump. Diabetes Care. 2007;30:549‐554. 17327319 10.2337/dc06-1755

[96] Vesco AT , Anderson BJ , Laffel LMB , Dolan LM , Ingerski LM , Hood KK . Responsibility sharing between adolescents with type 1 diabetes and their caregivers: importance of adolescent perceptions on diabetes management and control. J Pediatr Psychol. 2010;35:1168‐1177. 20444852 10.1093/jpepsy/jsq038 PMC2955833

[97] Ingerski LM , Anderson BJ , Dolan LM , Hood KK . Blood glucose monitoring and glycemic control in adolescence: contribution of diabetes‐specific responsibility and family conflict. J Adolesc Health. 2010;47:191‐197. 20638012 10.1016/j.jadohealth.2010.01.012 PMC2907244

[98] Barnard KD , Lloyd CE , Dyson PA , et al. Kaleidoscope model of diabetes care: time for a rethink? Diabet Med. 2014;31:522‐530. 24506524 10.1111/dme.12400

[99] Helgeson VS , Reynolds KA , Siminerio L , Escobar O , Becker D . Parent and adolescent distribution of responsibility for diabetes self‐care: links to health outcomes. J Pediatr Psychol. 2008;33:497‐508. 17848390 10.1093/jpepsy/jsm081 PMC3442285

[100] Harrison S , Stadler M , Ismail K , Amiel S , Herrmann‐Werner A . Are patients with diabetes mellitus satisfied with technologies used to assist with diabetes management and coping?: a structured review. Diabetes Technol Ther. 2014;16:771‐783. 25069057 10.1089/dia.2014.0062

[101] Dixon‐Woods M , Cavers D , Agarwal S , et al. Conducting a critical interpretive synthesis of the literature on access to healthcare by vulnerable groups. BMC Med Res Methodol. 2006;6:1‐13. 16872487 10.1186/1471-2288-6-35 PMC1559637

[102] NHS Digital . The National Diabetes Audit Insulin Pump Report 2015‐16. Leeds: NHS Digital; 2017. Page 3.

